# Establishment and application of a method of tagged-amplicon deep sequencing for low-abundance drug resistance in HIV-1

**DOI:** 10.3389/fmicb.2022.895227

**Published:** 2022-08-22

**Authors:** Yang Li, Leilei Han, Yanglan Wang, Xiaolin Wang, Lei Jia, Jingyun Li, Jingwan Han, Jin Zhao, Hanping Li, Lin Li

**Affiliations:** ^1^School of Basic Medical Sciences, Anhui Medical University, Hefei, Anhui, China; ^2^Department of Virology, State Key Laboratory of Pathogen and Biosecurity, Beijing Institute of Microbiology and Epidemiology, Beijing, China; ^3^School of Public Health, North China University of Science and Technology, Tangshan, Hebei, China; ^4^College of Life Science and Technology, Beijing University of Chemical Technology, Beijing, China; ^5^Shenzhen Center for Disease Control and Prevention, Shenzhen, Guangdong, China

**Keywords:** HIV-1, next-generation sequencing, minority drug resistance variants, assessment, standardization

## Abstract

In the latest HIV-1 global drug resistance report released by WHO, countries are advised to strengthen pre-treatment monitoring of drug resistance in AIDS patients. In this study, we established an NGS-based segmented amplification HIV-1 drug resistance mutation detection method. The *pol* region of HIV-1 was divided into three short fragments for NGS. The entire amplification and sequencing panel were more cost-effective and batched by using the barcode sequence corresponding to the sample. Each parameter was evaluated using samples with known resistance variants frequencies. The nucleotide sequence error rate, amino acid error rate, and noise value of the NGS-based segmented amplification method were both less than 1%. When the threshold was 2%, the consensus sequences of the HIV-1 NL4-3 strain were completely consistent with the Sanger sequences. This method can detect the minimum viral load of the sample at 10^2^ copies/ml, and the input frequency and detection frequency of HIV-1 resistance mutations within the range of 1%–100% had good conformity (R^2^ = 0.9963; R^2^ = 0.9955). This method had no non-specific amplification for Hepatitis B and C. Under the 2% threshold, the incidence of surveillance drug resistance mutations in ART-naive HIV-infected patients was 20.69%, among which NRTIs class resistance mutations were mainly.

## Introduction

The global HIV-1 drug resistance report released by WHO (2021) pointed out that more and more countries are on the verge of reaching the 10% pre-treatment drug resistance threshold for non-nucleoside reverse transcriptase inhibitors in the WHO releases HIV drug resistance report 2021. As the coverage of antiretroviral drugs expands and patient treatment experience increases, the prevalence of HIV resistance is expected to increase (Xie et al., 2021). The presence of drug-resistant HIV in the viral population, including the minor frequency drug resistance variants (Palmer et al., 2005), is known to compromise virological response to antiretroviral therapy (ART; Soeria-Atmadja et al., 2020). Minor resistant variants were associated with reduced virologic response and failure of ART (Halvas et al., 2010; Nicot et al., 2012). Low-frequency HIV-1drug resistance variants, particularly NNRTI resistance, were significantly associated with a dose-dependent increase in the risk of first-line ART virological failure (Gupta et al., 2014). If no measures are taken to deal with the problem of drug resistance, this may become a major obstacle to achieving the 90%–90%–90% control target. Once the virus develops drug resistance, the viral load will continue to increase, leading to the failure of ART treatment (Tachbele et al., 2021). If the problem of HIV-1 drug resistance cannot be effectively controlled, it may lead to the widespread of drug-resistant viruses and the emergence of multidrug-resistant (Guglielmetti et al., 2021).

The detection of HIV-1 drug resistance mutations often uses Sanger sequencing (SS) as the “gold standard” (Stranneheim and Lundeberg, 2012). However, SS generally detects variants accounting for greater than 10%–25% of the viral quasispecies (Grant et al., 2003; Chen et al., 2020), which is not sensitive enough to detect low-frequency resistance variants. To assess minor drug resistance mutations of HIV *in vivo*, single genome sequencing (SGS; McKinnon et al., 2011; Wang et al., 2014) and allele-specific real-time PCR (ASPCR; Hauser et al., 2015) were developed, but they cannot be applied to large-scale samples detection due to time-consuming and labor-intensive. In recent years, NGS is becoming a more common sequencing method that is widely used in the current genetic detection field (Gibson et al., 2020; Bendall et al., 2021; Parikh et al., 2021). NGS can detect low-abundance drug resistance mutations by sequencing the HIV-1 quasispecies (Ji et al., 2020). In HIV-1 drug resistance mutation detection, NGS has the advantages of high sensitivity, high data throughput, and parallel detection of large batches of samples. Nevertheless, there is currently no standardized evaluation system for NGS-based HIV drug resistance detection. The Sentosa SQ HIV genotyping assay (Tzou et al., 2018) is currently the only HIV-1 genotyping NGS detection method on the market that has been approved by the U.S. FDA. For the currently widely used NGS-based HIV drug resistance mutation detection method, a standardized evaluation system should be established.

In this study, we established an NGS-based HIV-1 drug resistance mutation detection method that allows surveillance of HIV-1 minor resistant variants in population. HIV strains with known drug resistance variants was used to construct sample to evaluate various parameters of the methods. And this method was used to detect minority drug resistance variants in newly diagnosed HIV-infected patients in Shenzhen from 2014 to 2015.

## Materials and methods

### Design of tagged-amplicon deep sequencing of fragmented pol region

**Figure 1:**
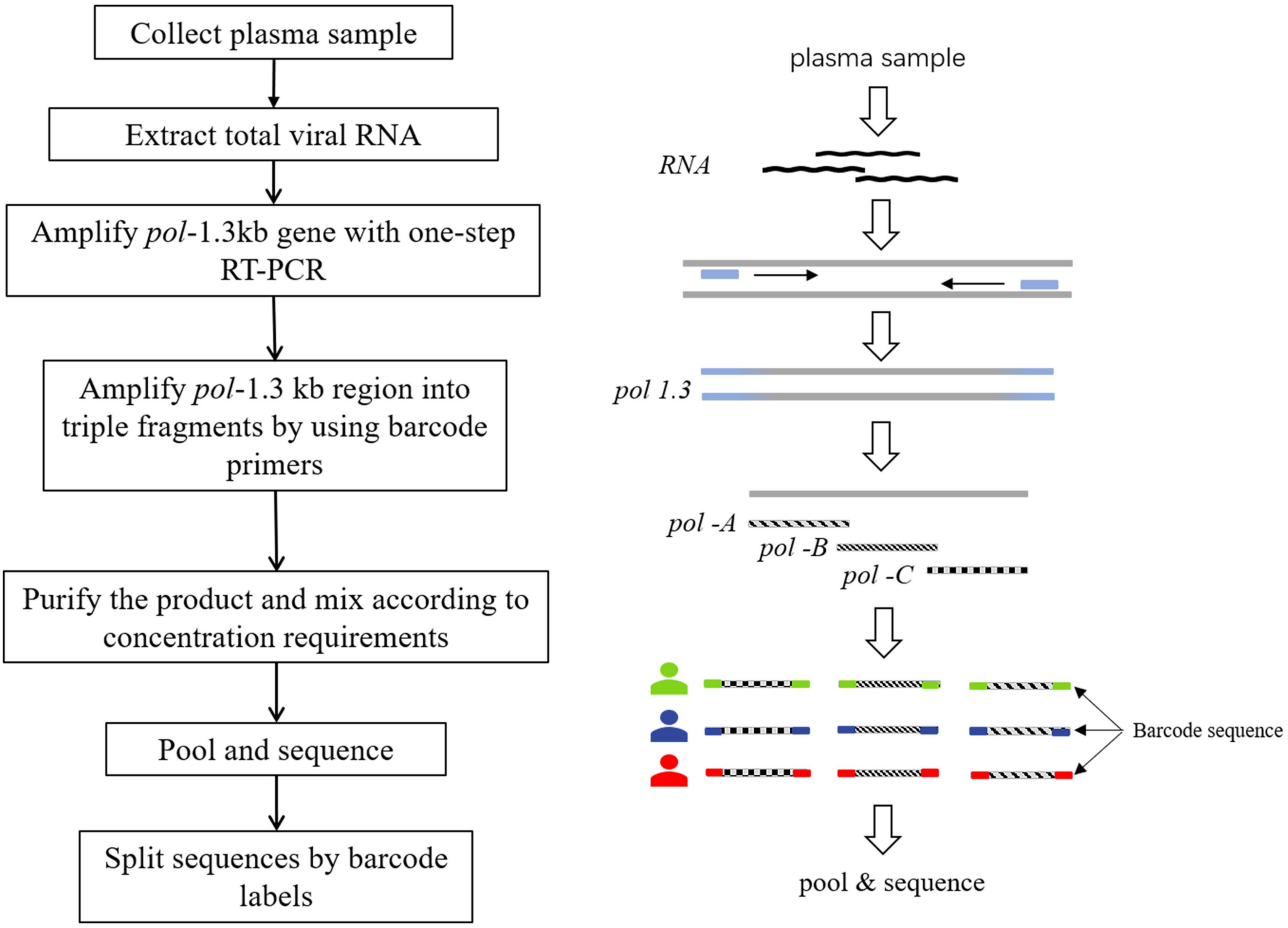
Tagged-amplicon deep sequencing strategy. HIV-1 *pol*-1.3kb gene was amplified with one-step RT-PCR. Subsequently, nested PCR was used to amplify pol-1.3kb region into 3 fragments, labeled as *pol*-A, *pol*-B, and *pol*-C. There was a small overlap at the junction of the three amplicons. The molecular barcode was attached to both ends of the amplicons. Barcodes were used to split data to distinguish different samples.

The segmented tagged-amplicon technology for HIV-1 drug resistance detection was based on the next-generation sequencing platform, and the short sequencing length was the main limitation of the NGS platform. To obtain amplicons suitable for NGS, the HIV-1 *pol* gene region covering the PR and first 226 codons of the RT was amplified in triple fragments with one-step RT-PCR (Takara, RR055A) and nested PCR (Takara, RR901A). The primer sequences used for amplification are shown in Supplementary Table 1. And the effective sequencing length was extended through paired-end sequencing. Each amplicon was approximately 400 bases in length, denoted *pol*-A, *pol*-B, and *pol*-C (nucleotide 2,169~2,583, 2,540~2,944, and 2,834~3,228 by using HXB2 as calibrator). There was a small overlap between the beginning and the end of these three amplicons. There is a small overlap between the start and end of these three amplicons, resulting in an overall amplification length of 1,060 bp. In addition, the barcode sequence was attached to the amplicons to tag different samples (Figure 1). P5/P7 tails were added at both ends of the amplified fragment to construct a high-throughput sequencing library. In a flowcell, amplicon fragment generated clusters by bridge PCR, and hundreds to thousands of amplified fragments were sequenced simultaneously. The amplicon fragments were sequenced on the Illumina sequencing platform using sequencing-by-synthesis sequencing.

### Study specimens and population

In order to evaluate and validate the HIV-1 drug resistance detection method, we developed a reference group for this study. Viral load of the wild-type HIV-1 NL4-3 strain (accession number AF003887) and the resistant mutant strains were determined using quantitative real-time PCR and then mixed the wild-type HIV-1 NL4-3 strain and the resistant mutant strains according to the viral load concentration to obtain targeted DRM frequencies of 1%, 2%, 10%, 30%, and 100%. The 187 reference specimens consisted of a known ratio of virus mixtures, HIV-1 NL4-3 strain, Hepatitis B clinical samples, Hepatitis C clinical samples, and HIV-1 positive clinical samples (Supplementary Table 1). The 190 reference samples consisted of known ratios of virus mixtures, HIV-1 NL4-3 strains, Hepatitis B clinical samples, Hepatitis C clinical samples, healthy human plasma samples (HIV-1-/HBV-/HCV-), and HIV-1 positive clinical samples (Supplementary Table 2). Hepatitis B and C plasma samples were provided by clinical patients and these plasma samples were negative after the rapid HIV-1 test.

All newly HIV-1 diagnosed cases in Shenzhen from 2014 to 2015 were enrolled in this study. All patients were antiretroviral-naïve. Blood samples were collected by Shenzhen Center for Disease Prevention and Control. All participants signed written informed consents before sample collection and their background information was collected. This study was reviewed and approved by the ethics committees of the Beijing Institute of Microbiology and Epidemiology.

### NGS-based HIVDR assay

For the NGS, after the mixed original sequencing results were obtained on the Illumina sequencing platform, the sequencing data of different samples were split by barcode sequence. Quality control is the first step in processing high-throughput sequencing data. FastQC^1^ was used for quality control of fastq files obtained from sequencing. Then, the fastq_1 and fastq_2 files obtained by paired-end sequencing were spliced to generate the sequencing data of each sample. Mothur (Schloss et al., 2009) was used to exclude poor-quality sequences from the sequencing data, and mafft (Katoh et al., 2019) was used to align the sequencing results with the reference sequence. HXB2 was used as a ruler to locate the target drug-resistant position. According to the mutations for drug resistance surveillance list of HIV drug resistance database of Stanford University^2^, the proportion of the mutations related to drug-resistant at that location was counted.

### SS-based HIVDR amplification and assay

The Sanger sequencing amplification process involved in this study was completed according to the following method. Roche High Pure Viral RNA Kit (REF:11858882001) was used to extract total viral RNA from plasma samples. The *pol* gene region (nucleotides positions 2,253–3,555, using HXB2 as the calibrator) spanning the protease gene and partial reverse transcriptase gene was amplified in the first round of nested reverse transcriptase-polymerase chain reaction (RT-PCR) using the One-Step RNA PCR Kit (Takara, RR055A). And the r-Taq kit (Takara, RR901A) was used in the additional PT-PCR. The primer sequences used for Sanger amplification are shown in Supplementary Table 3. Before sequencing, the PCR products were detected by 1% agarose gel electrophoresis and then subjected to direct DNA sequencing on an Applied Biosystems 3,730 Sequencer.

Sanger sequences were manually assembled in Contig Express software. The nucleotide where the secondary peak was at least 30% as high as the primary peak was counted as the degenerate bases using the International Union of Pure and Applied Chemistry (IUPAC) designations to obtain the fasta sequences. The fasta sequences were submitted to the HIV drug resistance database of Stanford University^3^ to generate a drug resistance report.

### NGS SDRMs

Due to the sensitivity of NGS to HIV low-abundance quasispecies populations, the setting of thresholds for deep sequencing data is important for reporting drug resistance variants (Kireev et al., 2018). At present, there is no accurate diagnostic value of minority drug resistance variants of HIV-1 in clinical practice. Studies have shown that the detection of NGS had a good linear relationship in the range of drug resistance mutation frequency from 1% to 100% (Lee et al., 2020). However, compared with the reporting threshold of 1%, the reporting threshold of 2% is not easy to introduce errors caused by PCR amplification and different high-throughput data analysis platforms (Chaillon et al., 2017), and has better robustness and repeatability (Becker et al., 2020; Parkin et al., 2020). Therefore, we chose a reporting threshold of 2% to describe resistance in HIV-infected individuals.

## Results

### The overall sequence error rate

**Figure 2:**
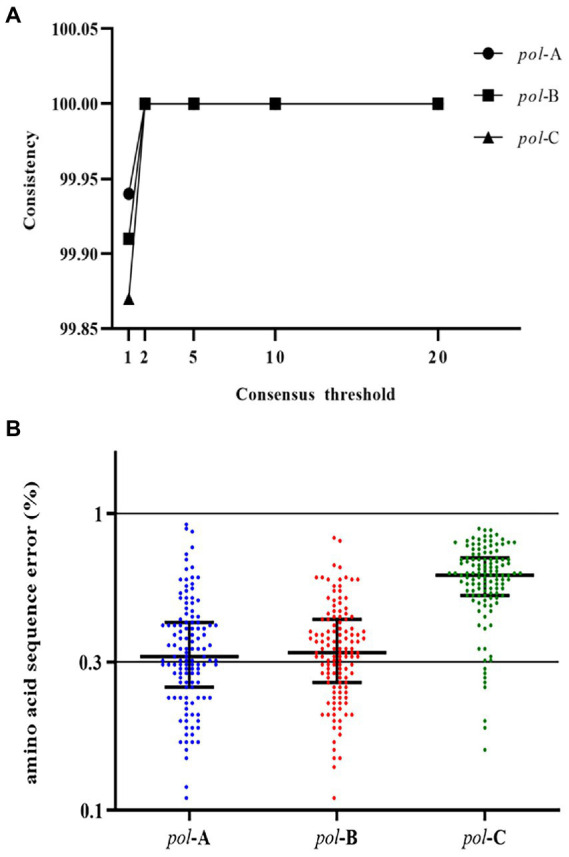
**(A)** Similarity between consensus sequences and SS sequence. Consensus sequences were generated with NGS data under thresholds of 1%, 2%, 5%, 10%, and 20%. Compared with the SS sequence, degenerate bases or incorrectly called bases were regarded as inconsistent bases. **(B)** The amino acid sequence error of the three fragments of the *pol* gene region. Compared with the known sequence, the inconsistent amino acids were all regarded as the wrongly called amino acids. The proportion of error amino acids was defined as the error rate of the amino acid sequence at that position.

The NGS sequencing results of the review team are presented in Supplementary Table 4. We wanted to measure the overall sequence error. The overall sequence error was defined as the errors in the entire experimental process of viral nucleic acid extraction, PCR amplification, NGS sequencing, and data processing and analysis. The HIV-1 NL4-3 strain with a known sequence was used for this measurement. We used the segmented amplification method to extract, amplify, and sequence the HIV-1 NL4-3 strain and measured the average frequency of erroneous nucleotide calls. We used the well-established RNA extraction method (Roche High Pure Viral RNA Kit) to improve the RNA recovery and quality of the HIV-1 NL4-3 strain, as well as the tagged amplicon deep sequencing method to amplify and sequence. And measured the average frequency of erroneous nucleotide calls. The error rate of nucleotide sequences we measured using viral RNA ranged from 0.006% to 0.945%, with an average of 0.164%. The consensus sequences generated under the threshold greater than 1% were completely consistent with the SS sequences (Figure 2A). The detection of HIV-1 resistance mutations was the identification of amino acids, so we measured the amino acid sequence error rate of the segmented amplification method. The results showed (Figure 2B) that the average amino acid sequence error rate of *pol*-A gene region was 0.37% (range: 0.11%–0.92%), *pol*-B was 0.36% (range: 0.11%–0.83%), and *pol*-C was 0.61% (range: 0.16%–0.89%). This suggested that the analysis error rate of our HIVDR detection method at the amino acid level was less than 1%.

### The lower limit of viral load

To determine the minimum viral load concentration that can be detected by our segmented amplification method, we used HIV-1 NI4-3 mutant strain as a single strain type sample, and wild-type HIV-1 NL4-3 strain and resistance mutant strain were mixed with a DRM frequency of 60% as a mixed strain type sample. Quantitative Real-time PCR was used to detect the viral load level of the samples. Each type of sample was diluted to a different viral load concentration, and each sample with a viral load concentration was divided into 5 evenly for viral nucleic acid extraction, amplification, and sequencing. We define a detection rate of less than 80% as a detection failure. It can be seen from the test results that our HIVDR detection method has a minimum viral load of 10^2^ copies/ml for samples composed of a single strain or a sample of mixed strains (Supplementary Table 5).

### Linear range and accuracy

**Figure 3:**
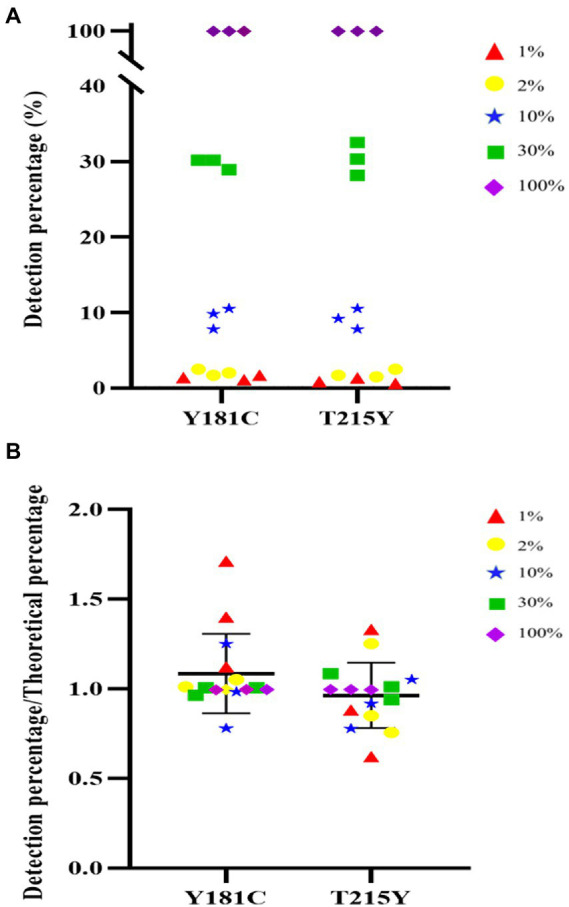
**(A)** Distribution of detect results at different frequencies. **(B)** The distribution of the ratio of the detection frequency to the theoretical frequency.

**Figure 4:**
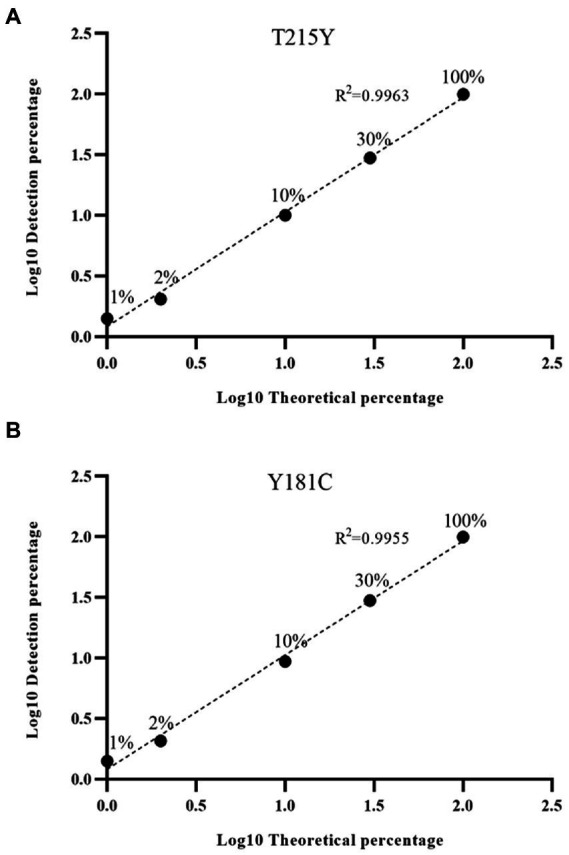
**(A)** Linear detection range of T215Y drug resistance mutation. In our detection range, the input frequency and detection frequency of Y181C and T215Y showed a good correlation. **(B)** Linear detection range of Y181C drug resistance mutation.

Next, we were interested in whether the HIVDR detection method was consistent with the input resistance frequency and the detection frequency. Samples with different DRM frequencies were used for viral nucleic acid extraction, amplification, and sequencing. The drug resistance mutations were reported from the high-throughput sequencing data through the above-mentioned analysis process. The detection results at different frequencies were plotted as scatter plots. And we calculated the ratio of the detection frequency to the theoretical frequency. The results of the ratio were distributed around 1, which also showed that the HIVDR detection method had good accuracy (Figure 3). The average of the resistance frequency of three parallel repetitions was calculated as the actual detection frequency, and a fitting curve was made with the input theoretical frequency. From all the tested frequencies, whether it was Y181C or T219Y, the detection frequency and the theoretical frequency had highly relevant. of compliance within the range of 1%–100% (R^2^ > 0.99), which proved that the segmented amplification method can accurately detect resistance mutations with a frequency of more than 1% (Figure 4).

### Precision

The coefficient of variation was used to express the reproducibility between three replicates within a batch. We calculated the intra-batch coefficient of variation based on the results of three replicates on mixed samples with different DRM frequencies. The coefficients of variation of Y181C and T215 were between 0.08%–23.6% and 0.04%–38.22%, respectively, (Supplementary Table 6). The accuracy of this detection method was the worst when the drug resistance mutation frequency was 1%. As the frequency of drug resistance mutation increased, the precision performance was better. It was not surprising that NGS was more stable in the detection of high-frequency resistance mutations due to the sensitivity of NGS detection and the deviations produced by the PCR process.

### Specificity and noise value

To verify the specificity of the segmented amplification method, we selected Hepatitis B and C virus for detection. Hepatitis B and C samples were provided by confirmed clinical patients, and these samples were HIV-1 negative after rapid HIV testing. The Hepatitis B and C samples were amplified using the segmented amplification method, and the results were all negative. It showed that the amplification method had good specificity to the HIV-1 virus, and the possibility of amplifying other virus sequences was low (Supplementary Table 7).

**Figure 5:**
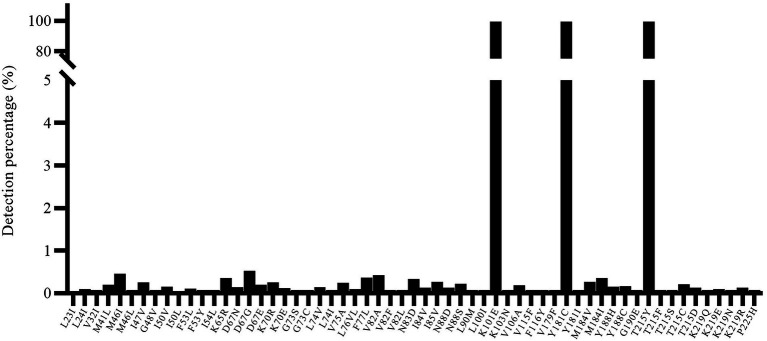
The specificity of known resistance mutations and noise value. The HIV-1 NL4-3 with K101E + Y181C + T215Y mutant strain was used for detection, and all three target mutations were detected. At the same time, there were some false positive mutations with a frequency of less than 1% (maybe introduced for PCR amplification).

In addition, we also used a well-characterized HIV-1 NL4-3 mutant (K101E + Y181C + T215Y) as input to verify the specificity of the segmented amplification method. The frequency of resistance mutation of three independent replicates was counted, and resistance mutations that only appear once will also be included, and the average of the reported frequency of resistance mutations that appeared multiple times will be taken (Figure 5). Judging from the HIV-1 NL4-3 (K101E + Y181C + T215Y) detection report, some ultra-low-frequency (<1%) false-positive results appeared in the results of the segmented amplification method. The detection specificity of the target mutation can reach 100%.

### Method evaluation

**Figure 6:**
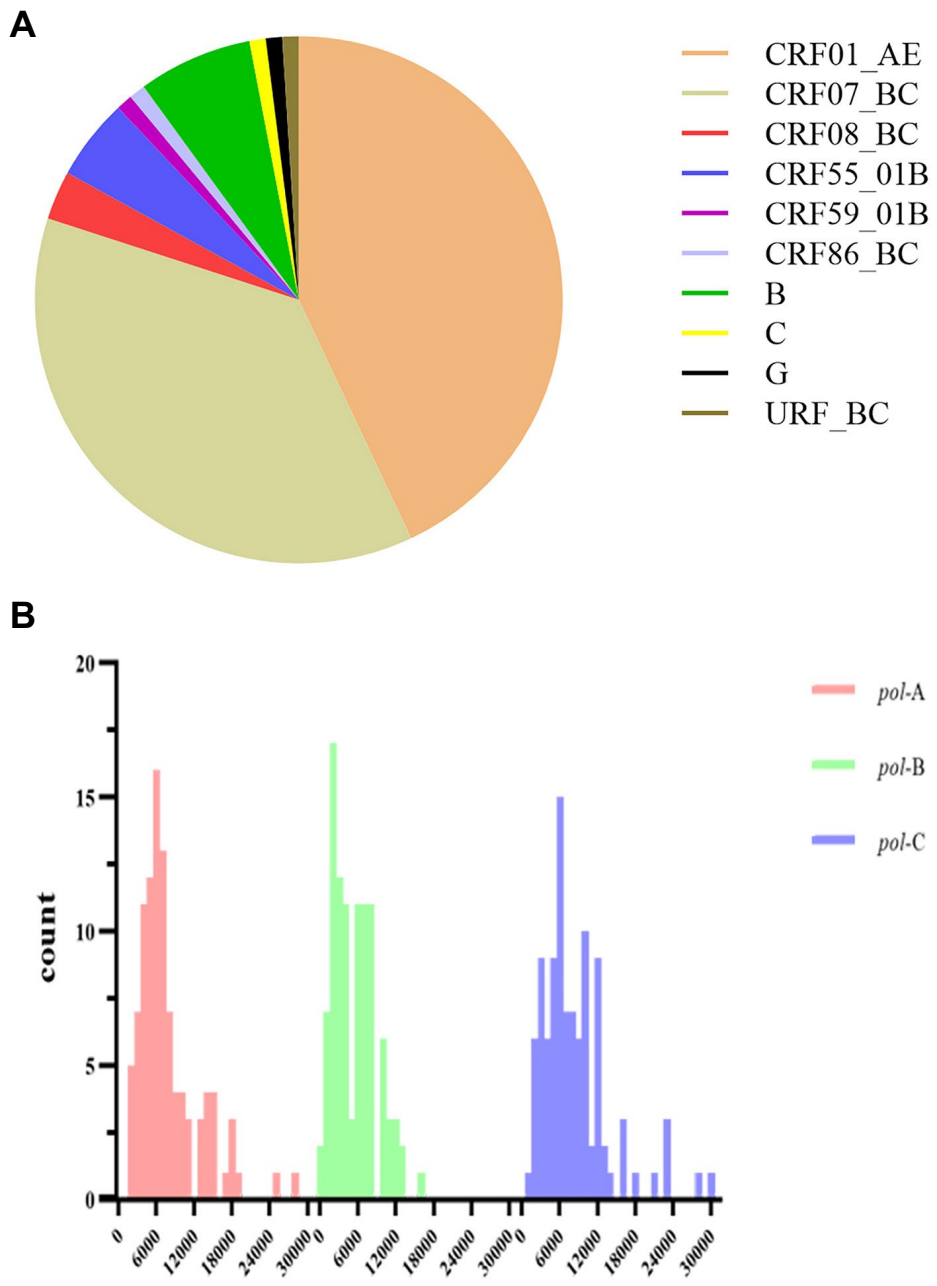
**(A)** Subtype distribution of HIV-1 clinical samples. **(B)** The distribution of reads in the deep sequencing data of HIV-1 clinical samples. After sequence quality control, the poor-quality sequences were filtered. Different colors represented the distribution of reads in the three segments in the *pol* gene region. The reads of *pol*-A, *pol*-B, and *pol*-C were respectively concentrated in 6,000 (range:1,694–28,069), 2,000 (range:389–16,008), and 6,000 (range:1,373–30,018).

One hundred plasma samples of HIV-1 clinical patients were randomly trained and detected using the In-house method and segmented amplification method. The demographic characteristics of HIV-1 clinical samples are shown in Supplementary Table 8. The reads of deep sequencing data after quality control were mostly distributed around 6,000 sequences (Figure 6). A total of 10 subtypes were included in all samples. According to the detection results of surveillance drug resistance mutations (SDRMs), whether it was analyzed using our NGS-sequence analysis panel or HyDRA, the segmented amplification method can detect all the drug resistance mutations reported by Sanger sequencing. In addition, the NGS method reported an SDRM that had not been detected by SS, which indicated that the segmented amplification method had higher sensitivity than the traditional in-house method (Table 1).

**Table 1:** Comparison of the detection results of the three detection methods.

Sample number	In-house	NGS-sequence analysis panel^a^	HyDRA^a^
LS10185	Y181C	Y181C-41.16%	Y181C-39.82%
	K219N	K219N-50.51%	K219N-49.94%
LS10202		D67N-62.28%	D67N-59.10%
LS10221	M41L	M41L-23.09%	M41L-29.12%
LS10407	K103N	K103N-98.62%	K103N-98.53%
LS10564	Y181C	Y181C-37.04%	Y181C-27.21%

a The percentage indicated the reported frequency of the resistant mutation.

**Figure 7:**
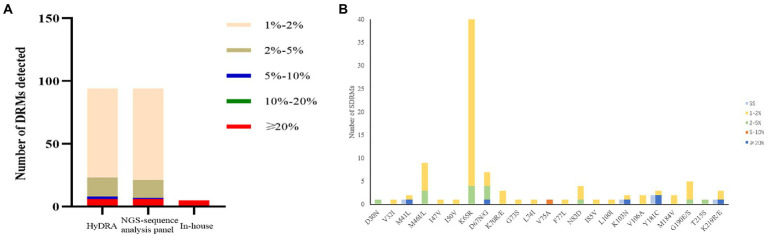
**(A)** The number of DRMs detected by the three detection methods. The range of the interval in the figure was inclusive of the left-side value, and the right-side value was not included. **(B)** Comparison of HIV-1 SDRMs detected by Sanger sequencing and NGS-sequence analysis panel at different detection thresholds.

Our NGS-sequence analysis panel and HyDRA were used to detect drug resistance mutations in NGS sequencing data, and the SDRMs with a frequency of more than 1% were counted (Figure 7). Due to the difference in algorithms, it was not surprising that the two methods have slight differences in the number of low-frequency SDRMs. The calculation results of McNemar’s test proved the accuracy of our NGS-sequence analysis panel for SDRM detection (*p* = 0.972).

### HIV-1 samples detection

All samples were amplified by PT-PCR using a segmented amplification method based on next-generation sequencing methods. Excluding factors such as insufficient plasma samples and failure of viral nucleic acid extraction, a total of 1,755 samples successfully completed the amplification and next-generation sequencing process. Among the samples for which sequencing results were obtained, 15 samples were excluded due to poor sequencing quality, and the remaining 1,740 samples obtained good sequencing results. The proportion of samples with all three segments of *pol*-A, *pol*-B, and *pol*-C positive was 99.15%.

A total of 1,740 participants with demographic data and NGS sequences. General characteristics of the study subjects are shown in Table 2. The age of the patients was mainly distributed between 20 and 50 years old. Most individuals were men (86.03%), single (54.25%), and Han Population (92.13%). The dominant transmission route was heterosexual contact (63.68%). The education level of junior high school and above accounted for 90.52%. CRF01_AE (37.13%) subtype was predominant, followed by CRF07_BC (38.22%) and CRF55_01B (9.54%).

**Table 2:** Demographic characteristics of HIV-infected persons in Shenzhen, 2014–2015.

Characteristics	Case number and percentage, *n* (%)
Total	1,740
**Age (years old)**	
≤20	7 (0.4%)
21–30	403 (23.16%)
31–40	676 (38.85%)
41–50	412 (23.68%)
>50	242 (13.91%)
Gender	
Male	1,497 (86.03%)
Female	244 (14.02%)
**Route of transmission**	
Homosexual	549 (31.55%)
Heterosexual	1,108 (63.68%)
Injecting drug using	55 (3.16%)
Unknown/other	28 (1.61%)
**Ethnic group**	
Han	1,603 (92.13%)
Others	137 (7.87%)
**Marital status**	
Single	944 (54.25%)
Married or living with partner	560 (32.18%)
Divorced or widowed	227 (13.05%)
Unknown	9 (0.52%)
**Education**	
Illiterate	18 (1.03%)
Primary school	147 (8.45%)
Junior middle school	690 (39.66%)
Senior school and technical secondary school	501 (28.79%)
College and above	384 (22.07%)
**Subtype**	
CRF01_AE	646 (37.13%)
CRF07_BC	665 (38.22%)
CRF55_01B	166 (9.54%)
B	91 (5.23%)
Others	172 (9.89%)

**Figure 8:**
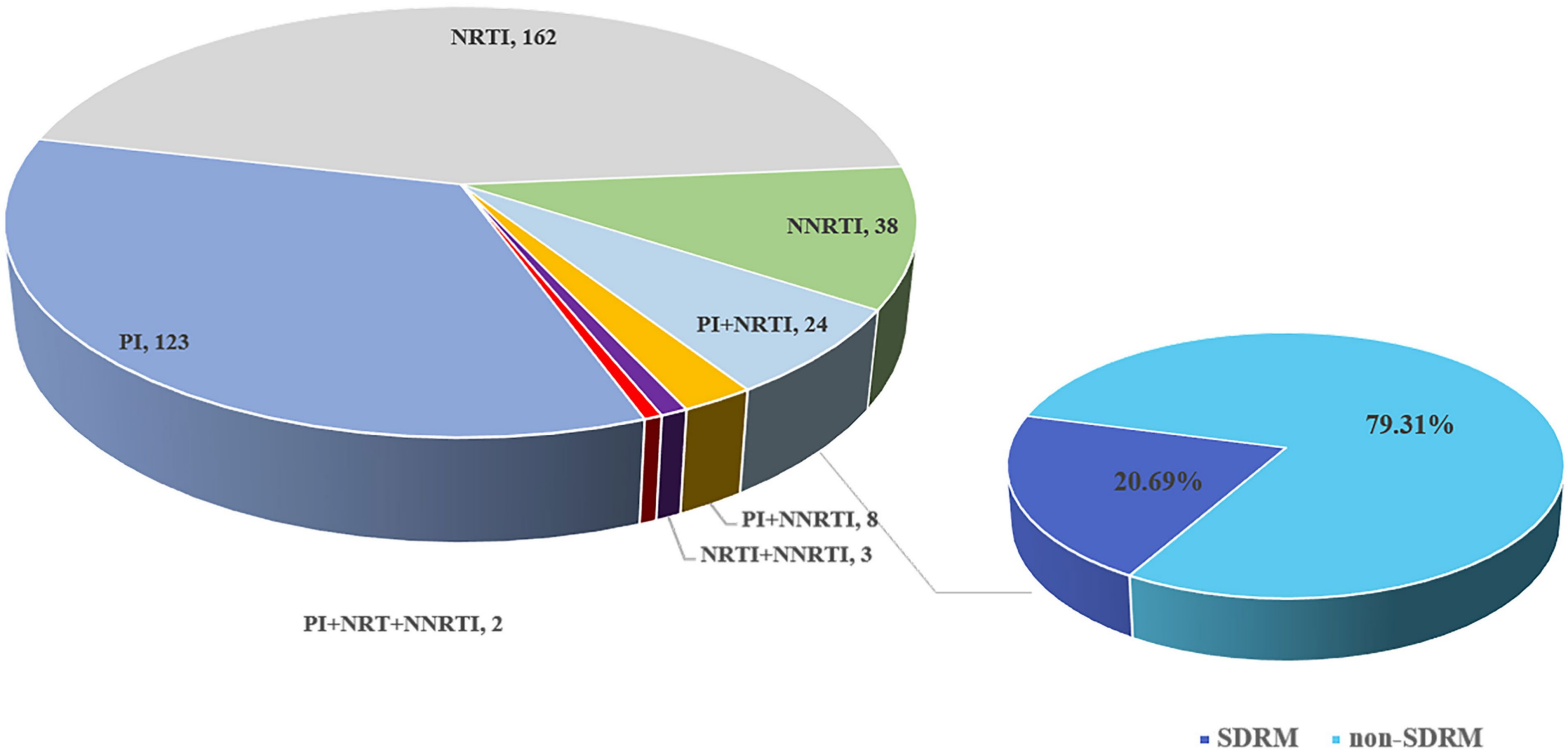
Composition and distribution of SDRM in newly diagnosed HIV-1 samples in Shenzhen from 2014 to 2015.

According to the mutations for drug resistance surveillance in the Stanford Drug Resistance Database, a total of 360 out of 1,740 patients contained SDRMs at the 2% threshold (Figure 8). The NRTIs resistance mutations were the most prevalent (48.14%), followed by PIs (39.3%) and NNRTIs (12.56%). Thirty-five strains (2.01%) contain dual-class mutations (PI + NRTI in 24 cases, PI + NNRTI in 8 cases, and NRTI + NNRTI in 3 cases) and 2 cases contain triple-class variants. For NRTIs, the most common mutation was K65R and for PIs there were M46I + N88S. the most frequent mutations related to NNRTIs were G190E.

## Discussion

Due to the unparalleled advantages of NGS, it is not surprising that NGS is used for HIV drug resistance mutation detection. In this study, a new amplification method based on next-generation sequencing was established to detect the minority resistance variants in HIV-infected patients. This method was based on the Illumina sequencing platform and used PCR amplicons for library construction. Compared with Sanger sequencing, NGS has higher detection sensitivity and automated data flow, and NGS has extremely high sensitivity for less than 20% of minority resistance variants. On the basis of Sanger sequencing to detect the existence of “yes” or “no” drug resistance variants, NGS adds more information about the frequency of variants.

The evaluation of the detection method is an important part of the whole method establishment. Many previous studies often used different data analysis platforms to analyze NGS sequences, and integrated the reports of each platform to finally determine the results of drug resistance detection (Lee et al., 2020). Different from previous studies, we used HIV strains with known resistance variants to complete the evaluation of NGS detection. The introduction of virus strains with known drug-resistant frequencies made the entire detection process completely simulate the amplification and detection process of actual samples, which more truly reflected the accuracy of NGS in detecting species at drug-resistant variants. An overall assessment of the actual errors introduced in the entire workflow from viral nucleic acid extraction to resistance site reporting was completed.

At present, the application of high-throughput sequencing to HIV drug resistance detection is still mainly in the laboratory research stage, and it is rarely used in clinical detecting. The clinical relationship between HIV minority resistance variants and disease is still inconclusive, but it has been documented that minority resistance variants may lead to the failure of HIV virological treatment (Gupta et al., 2014; Hikichi et al., 2021). Population-based studies have proven that NGS can detect a large proportion of low-level variants, while traditional Sanger sequencing cannot detect (Baxter et al., 2021; Tekin et al., 2021). Similarly, the same conclusion was obtained in our study. In the detection of the same samples, NGS outperformed SS in terms of detection accuracy and sensitivity. Compared with SS, NGS can detect more low-frequency resistance mutations. This suggests that the prevalence of resistance in previous reports may be underestimated. Low-frequency resistance mutations could have significant consequences for clinical outcomes (Li et al., 2011; Kyeyune et al., 2016). In our detection of HIV samples in Shenzhen, we found that there were a large number of low-abundance NRTIs resistance variants in the ART-naive HIV population, which may be related to the first-line antiviral drugs used in our country. This suggests that pre-treatment drug resistance testing before initiating ART in newly diagnosed HIV/AIDS patients may be necessary.

Although our evaluation indicators showed that the segmented amplification method can perform reliable quantitative analysis in the range of 1–100%, we do not recommend reporting resistance sites less than 2%. This is also consistent with the recommendations provided by the current research (Avila-Ríos et al., 2016; Lee et al., 2020). When the frequency was lower than 2%, the incidence of false positives increased, as well as the possibility of cross-contamination and PCR reaction deviation (Ávila-Ríos et al., 2020). At present, the optimal threshold for NGS detection of clinical drug resistance mutations is still inconclusive. By strengthening the standardization of NGS operating procedures and data analysis in drug resistance detecting, NGS is expected to become a new standard method for HIV drug resistance genotyping (Noguera-Julian et al., 2020).

It is undeniable that there were still some limitations in this study. The mutations contained in the HIV NL4-3 mutant used in this study were all reverse transcriptase resistance mutations, and no protease mutations were involved. In this method, only one high-throughput sequencing platform was used to establish and evaluate the drug resistance detection method, and the differences in sequencing errors among various high-throughput sequencing platforms were not included in the study. And samples at all frequencies between 1% and 100% were not included in the method evaluation.

In conclusion, we have established a method of tagged-amplicon deep sequencing for low-abundance drug resistance in HIV-1 and had completed the evaluation for the detection of HIV-1 drug resistance mutations. It provided some references for other studies using NGS and provided evaluation criteria for NGS technology to be used in HIV-1 clinical drug resistance detecting.

## Data availability statement

The original contributions presented in the study are included in the article/Supplementary material, further inquiries can be directed to the corresponding authors.

## Ethics statement

The studies involving human participants were reviewed and approved by the Beijing Institute of Microbial Epidemiology. Written informed consent to participate in this study was provided by the participants' legal guardian/next of kin. Written informed consent was obtained from the individual(s), and minor(s)’ legal guardian/next of kin, for the publication of any potentially identifiable images or data included in this article.

## Author contributions

LL, HL, and YL conceived the study. YL and LH conducted this study. YW provided help for data analysis. XW, LJ, JL, and JH provided guidance and help for the operation of the experiment. LL and HL provided guidance and reviewed the manuscript. JZ completed the sample collection. All authors contributed to the article and approved the submitted version.

## Funding

This study was supported by the National Key Research and Development Program of China (2020YFA0907000), National Natural Science Foundation of China (NSFC; 81773493, 31800149, and 31900157), and the State Key Laboratory of Pathogen and Biosecurity (AMMS; SKLPBS2103 and SKLPBS2118).

## Conflict of interest

The authors declare that the research was conducted in the absence of any commercial or financial relationships that could be construed as a potential conflict of interest.

## Publisher’s note

All claims expressed in this article are solely those of the authors and do not necessarily represent those of their affiliated organizations, or those of the publisher, the editors and the reviewers. Any product that may be evaluated in this article, or claim that may be made by its manufacturer, is not guaranteed or endorsed by the publisher.

## Footnotes

^1^ https://www.bioinformatics.babraham.ac.uk/projects/fastqc/

^2^ https://cms.hivdb.org/prod/downloads/resistance-mutation-handout/resistance-mutation-handout.pdf

^3^ https://hivdb.stanford.edu/hivdb/by-sequences/
