# Supplementary material for: Establishment and application of a method of tagged-amplicon deep sequencing for low-abundance drug resistance in HIV-1
Source: Front Microbiol. 2022 Aug 22;13:895227. doi: 10.3389/fmicb.2022.895227 (PMC9444182; doi:10.3389/fmicb.2022.895227)
Supplement: Supplementary file 1 [file Data_Sheet_1.docx]

**Supplementary Tables:**

**Table S1** **Amplification primers.**

|  | **Primer name** | **Sequence（5' to 3'）** | **Location on HXB2** |
| --- | --- | --- | --- |
| The first round of nested-PCR | DR-1 | TTGGAAATGTGCTAAGGAAGGAC | 2028-2050 |
|  | DR-2 | CACTCTCTGACCTGCTATCATCAT | 3529-3509 |
|  | DR-3 | CAGAGCGAACTGCCCCACCA | 2147-2166 |
|  | DR-4 | CTGCAATTTCTAGCTCTGCTTC | 3462-3441 |
| The second round of nested-PCR | *pol*-A_For | CAGAGCCAACATGCCCACCA | 2147-2166 |
|  | *pol*-A_Rev | GGGCCATCCGATCCTGGCTT | 2586-2605 |
|  | *pol*-B_For | GTTGACTAAGGTAGGTTGCAC | 2519-2539 |
|  | *pol*-B_Rev | CTGGTTCATTRTTKRTACTAGGT | 2945-2970 |
|  | *pol*-C_For | TTYTGGGARGTYCARYTAGTACC | 2808-2833 |
|  | *pol*-C_Rev | AGTTCATATGCCATCCAAAG | 3231-3250 |

**Table S2** **Amplification primers in Sanger sequencing.**

|  | **Primer name** | **Sequence（5' to 3'）** | **Location on HXB2** |
| --- | --- | --- | --- |
| The first round of nested-PCR | DR-1 | TTGGAAATGTGCTAAGGAAGGAC | 2028-2050 |
|  | DR-2 | CACTCTCTGACCTGCTATCATCAT | 3529-3509 |
|  | DR-3 | CAGAGCGAACTGCCCCACCA | 2147-2166 |
|  | DR-4 | CTGCAATTTCTAGCTCTGCTTC | 3462-3441 |
| The second round of nested-PCR | DR-5 | CAGATCCAACAGGCCTACCA | 2147-2166 |
|  | DR-6 | CTGCTAGTTCTCGCTCTCTTC | 3441-3462 |
|  | DR-7 | CTCCAATACTAATTGTGCTTC | 3441-3462 |

|  | **Panel** | **Description** | **Frequency** | **Replicates** | **Viral Load (copies / mL)** | **Known DRMs** |
| --- | --- | --- | --- | --- | --- | --- |
| HIV +/DR- | Amino acid sequence error | HIV-1 NL4-3 virus | 0% | 3 | 3.09E+04 | N/A |
| HIV-1 +/DR+ | Lower limit of viral load | simple | 100% | 5 | 6.87E+05 | K101E Y181C T215Y |
|  |  |  |  | 5 | 6.31E+04 |  |
|  |  |  |  | 5 | 2.81E+03 |  |
|  |  |  |  | 5 | 2.32E+02 |  |
|  |  |  |  | 5 | 5.73E+01 |  |
|  |  | mix | 60% | 5 | 1.15E+06 |  |
|  |  |  |  | 5 | 1.48E+05 |  |
|  |  |  |  | 5 | 4.43E+03 |  |
|  |  |  |  | 5 | 9.78E+01 |  |
|  |  |  |  | 5 | 3.41E+01 |  |
|  | Accuracy | HIV-1 NL4-3 mutant | 1% | 3 | 9.55E+04 | Y181C T215Y |
|  |  |  | 2% | 3 | 6.19E+04 |  |
|  |  |  | 10% | 3 | 5.16E+03 |  |
|  |  |  | 30% | 3 | 2.32E+05 |  |
|  |  |  | 100% | 3 | 1.87E+05 |  |
|  | Noise value | HIV-1 NL4-3 mutant | 100% | 3 | 6.94E+03 | K101E Y181C T215Y |
| HIV-1 -/DR- | Specificity | Hepatitis B (4 samples) | N/A | 2 | 6.19E+04 | N/A |
|  |  | Hepatitis C (4 samples) | N/A | 2 | 7.08E+05 | N/A |
| Control group | Negative control | HIV-1 -/HBV -/HCV - | N/A | 3 | N/A | N/A |
| HIV-1 + drug naïve | Method evaluation | HIV-1 cases (100 samples) | unknown | -- | -- | unknown |

**Table S3** **Specimen composition.**

**Table S4** **NGS sequencing metrics**

| **Panel** | **Replicates** | ***pol* region fragment** | **Number of reads** | **Q30(%)** |
| --- | --- | --- | --- | --- |
| Amino acid sequence error | 1 | *pol-A* | 830 | 98.00 |
|  |  | *pol-B* | 8490 | 99.32 |
|  |  | *pol-C* | 17744 | 98.98 |
|  | 2 | *pol-A* | 1970 | 98.92 |
|  |  | *pol-B* | 6607 | 99.20 |
|  |  | *pol-C* | 2060 | 98.52 |
|  | 3 | *pol-A* | 2631 | 98.66 |
|  |  | *pol-B* | 509 | 99.81 |
|  |  | *pol-C* | 7513 | 99.13 |
| Accuracy | 1%_1 | *pol-A* | 1134 | 99.69 |
|  |  | *pol-B* | 7586 | 98.38 |
|  |  | *pol-C* | 10213 | 99.08 |
|  | 1%_2 | *pol-A* | 3369 | 98.40 |
|  |  | *pol-B* | 9559 | 98.83 |
|  |  | *pol-C* | 17699 | 99.15 |
|  | 1%_3 | *pol-A* | 7238 | 98.66 |
|  |  | *pol-B* | 7803 | 99.37 |
|  |  | *pol-C* | 8173 | 98.96 |
|  | 2%_1 | *pol-A* | 2069 | 99.00 |
|  |  | *pol-B* | 13592 | 98.32 |
|  |  | *pol-C* | 14289 | 98.25 |
|  | 2%_2 | *pol-A* | 1461 | 98.98 |
|  |  | *pol-B* | 7891 | 98.81 |
|  |  | *pol-C* | 19770 | 99.14 |
|  | 2%_3 | *pol-A* | 6419 | 98.75 |
|  |  | *pol-B* | 11628 | 99.15 |
|  |  | *pol-C* | 10069 | 98.21 |
|  | 10%_1 | *pol-A* | 5952 | 98.99 |
|  |  | *pol-B* | 6815 | 99.24 |
|  |  | *pol-C* | 1119 | 98.36 |
|  | 10%_2 | *pol-A* | 3227 | 98.48 |
|  |  | *pol-B* | 5900 | 99.10 |
|  |  | *pol-C* | 6260 | 98.18 |
|  | 10%_3 | *pol-A* | 1054 | 99.01 |
|  |  | *pol-B* | 1612 | 99.21 |
|  |  | *pol-C* | 3340 | 98.82 |
|  | 30%_1 | *pol-A* | 895 | 98.35 |
|  |  | *pol-B* | 5103 | 99.25 |
|  |  | *pol-C* | 3299 | 99.13 |
|  | 30%_2 | *pol-A* | 1061 | 99.20 |
|  |  | *pol-B* | 3803 | 98.90 |
|  |  | *pol-C* | 1675 | 98.64 |
|  | 30%_3 | *pol-A* | 5917 | 98.63 |
|  |  | *pol-B* | 4288 | 98.84 |
|  |  | *pol-C* | 2957 | 98.40 |
|  | 100%_1 | *pol-A* | 1367 | 98.83 |
|  |  | *pol-B* | 9559 | 99.15 |
|  |  | *pol-C* | 17699 | 98.66 |
|  | 100%_2 | *pol-A* | 7439 | 98.92 |
|  |  | *pol-B* | 7803 | 99.37 |
|  |  | *pol-C* | 8173 | 98.96 |
|  | 100%_3 | *pol-A* | 3499 | 98.85 |
|  |  | *pol-B* | 13592 | 98.98 |
|  |  | *pol-C* | 14289 | 98.81 |
| Noise value | 1 | *pol-A* | 2149 | 99.11 |
|  |  | *pol-B* | 7586 | 99.69 |
|  |  | *pol-C* | 10213 | 98.38 |
|  | 2 | *pol-A* | 1367 | 98.83 |
|  |  | *pol-B* | 9559 | 99.15 |
|  |  | *pol-C* | 17541 | 98.66 |
|  | 3 | *pol-A* | 2107 | 99.19 |
|  |  | *pol-B* | 4090 | 99.16 |
|  |  | *pol-C* | 3753 | 98.43 |

**Table S5 Positive rate of lower limit of viral load.**

| **Sample type** | **Viral Load (copies / mL)** | **Positive rate** |
| --- | --- | --- |
| Simple | 10^5^ | 100% |
|  | 10^4^ | 100% |
|  | 10^3^ | 100% |
|  | 10^2^ | 100% |
|  | 10^1^ | 60% |
| Mix | 10^6^ | 100% |
|  | 10^5^ | 100% |
|  | 10^3^ | 100% |
|  | 10^2^ | 100% |
|  | 10^1^ | 20% |

**Table S6 Coefficient of variation within batches of different mutations.**

| **HIV-1 DRM (%)** | **Coefficient of variation (CV, %)** | |
| --- | --- | --- |
|  | Y181C | T215Y |
| 1 | 23.6 | 38.22 |
| 2 | 19.67 | 24.65 |
| 10 | 15.1 | 14.92 |
| 30 | 2.44 | 7.2 |
| 100 | 0.08 | 0.04 |

**Table S7 Background information on Hepatitis B and C samples.**

| **Sample type** | **Gender** | **Age (years)** |
| --- | --- | --- |
| Hepatitis B virus | female | 43 |
| Hepatitis B virus | male | 59 |
| Hepatitis B virus | male | 47 |
| Hepatitis B virus | female | 60 |
| Hepatitis C virus | female | 86 |
| Hepatitis C virus | female | 75 |
| Hepatitis C virus | female | 61 |
| Hepatitis C virus | male | 39 |

**Table S8 Demographic characteristics of 100 samples.**

| **Characteristics** | **Case number and percentage, n (%)** |
| --- | --- |
| **Total** | 100 |
| **Age (years old)** |  |
| ≤30 | 9 (9%) |
| 31-50 | 71 (71%) |
| >50 | 20 (20%) |
| **Gender** |  |
| Male | 87 (87%) |
| Female | 13 (13%) |
| **Route of transmission** |  |
| Homosexual | 11 (11%) |
| Heterosexual | 85 (85%) |
| Injecting drug using | 4 (4%) |
| **Ethnic group** |  |
| Han | 94 (94%) |
| Others | 6 (6%) |
| **Marital status** |  |
| Single | 47 (47%) |
| Married or living with partner | 40 (40%) |
| Divorced or widowed | 13 (13%) |
| **Education** |  |
| Illiterate and primary school | 13 (13%) |
| Junior middle school | 44 (44%) |
| Senior school and technical secondary school | 43 (43%) |
